# Species-Specific Functional Regions of the Green Alga Gamete Fusion Protein HAP2 Revealed by Structural Studies

**DOI:** 10.1016/j.str.2018.09.014

**Published:** 2019-01-02

**Authors:** Eduard Baquero, Juliette Fedry, Pierre Legrand, Thomas Krey, Felix A. Rey

**Affiliations:** 1 Institut Pasteur, Unité de Virologie Structurale, 25-28 Rue du Docteur Roux, 75724 Paris Cedex 15, France; 2 CNRS UMR 3569, 25-28 Rue du Docteur Roux, 75724 Paris Cedex 15, France; 3 Synchrotron SOLEIL, L'Orme des Merisiers, 91192 Gif-sur-Yvette, France

**Keywords:** fertilization, gamete fusion, eukaryotic reproduction, structural biology, membrane fusion, green algae, *Chlamydomonas reinhardtii*, last eukaryotic common ancestor, protein evolution, virus-cell gene exchanges in evolution

## Abstract

The cellular fusion protein HAP2, which is structurally homologous to viral class II fusion proteins, drives gamete fusion across several eukaryotic kingdoms. Gamete fusion is a highly controlled process in eukaryotes, and is allowed only between same species gametes. In spite of a conserved architecture, HAP2 displays several species-specific functional regions that were not resolved in the available X-ray structure of the green alga *Chlamydomonas reinhardtii* HAP2 ectodomain. Here we present an X-ray structure resolving these regions, showing a target membrane interaction surface made by three amphipathic helices in a horseshoe-shaped arrangement. HAP2 from green algae also features additional species-specific motifs inserted in regions that in viral class II proteins are critical for the fusogenic conformational change. Such insertions include a cystine ladder-like module evocative of EGF-like motifs responsible for extracellular protein-protein interactions in animals, and a mucin-like region. These features suggest potential HAP2 interaction sites involved in gamete fusion control.

## Highlights

• Unprecedented organization of amphipathic α helices in the algal HAP2 fusion loops
• An inserted EGF-like motif suggests a potential algal-specific fusion control site
• An adjacent mucin-like region potentially modulates algal-specific interactions
• Inter-chain stem/domain II interactions stabilize the post-fusion hairpin conformation

## Graphical Abstract

**Figure:**
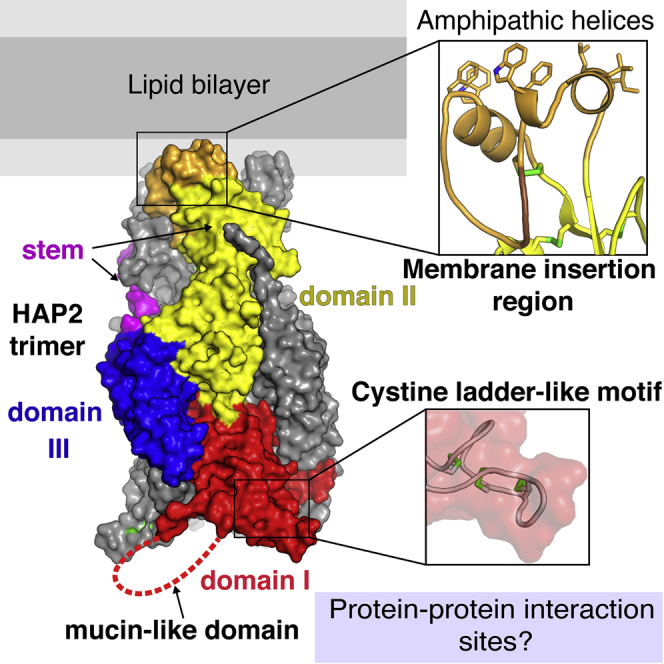

## Teaser

Baquero et al. report the structure of the HAP2 gamete fusion protein of the *Chlamydomonas reinhardtii* alga, describing key elements required to bridge two opposite-type gametes and drive their fusion. Mutagenesis of these elements confirmed their membrane-interaction function. Additional specific HAP2 protein-protein interaction sites are described as potential fusion controls.

## Introduction

Cell-cell fusion is ubiquitous across eukaryotes. It is required for fertilization, where two gametes of opposite type merge together to form a zygote (Georgadaki et al., 2016). During the development of multicellular organisms, the formation of syncytial tissues also occurs via fusion of somatic cells (Zito et al., 2016). The molecular mechanism driving the merger of lipid bilayers has been studied extensively in the case of intracellular fusion events, such as the fusion of transport vesicles (Jahn and Scheller, 2006, Martens and McMahon, 2008, Sudhof and Rothman, 2009, Wickner and Schekman, 2008) or the homotypic fusion of intracellular organelles (Farmer et al., 2018, Hu and Rapoport, 2016). In contrast, relatively little is known about extracellular fusion (Hernandez and Podbilewicz, 2017, Sampath et al., 2018). The only well-characterized extracellular fusion process is that of enveloped viruses, which fuse the viral lipid bilayer either with the plasma membrane or with an endosome from the luminal side (Harrison, 2015). HAP2/GCS1 is the first bona fide gamete fusion protein identified. Discovered in flowering plants (Johnson et al., 2004, Mori et al., 2006), subsequent studies in the green alga *Chlamydomonas reinhardtii* and the malaria organism *Plasmodium* showed that it was essential for bilayer merger (Liu et al., 2008). The presence of HAP2 in organisms across the main branches of eukaryotes (Cole et al., 2014, Ebchuqin et al., 2014, Kawai-Toyooka et al., 2014, Steele and Dana, 2009) suggests that it is likely an ancestral gamete fusogen already present at the origin of eukaryotic life (Wong and Johnson, 2010). HAP2 is present exclusively in male gametes in most of the organisms where it was identified—or in minus type gametes in the case of isogamous organisms such as algae. The gamete fusion process has been studied in highest detail in *C. reinhardtii*, where HAP2 was shown to localize to the “mating structure”, a projecting organelle at the plasma membrane located in between the two flagella of the *minus* gametes (Pan et al., 2003).

**Figure 1:**
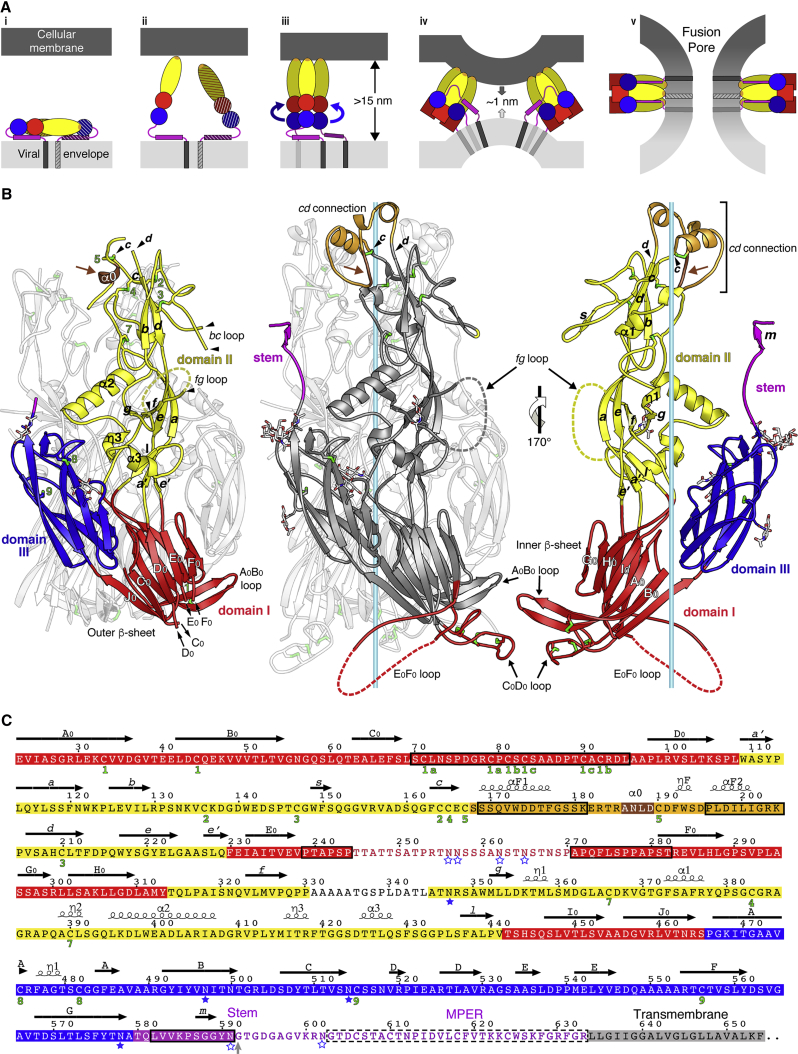
The New Structure Reveals Functional Species-Specific Regions of HAP2 (A) Diagram illustrating the rearrangement of viral class II fusion proteins to catalyze membrane fusion. The three domains are colored red, yellow, and blue (for domains I, II, and III, respectively), the fusion loop orange, the stem magenta, and the TM segment gray. Viral and cellular membranes are in light and dark gray, respectively. (i) Prefusion arrangement, with the fusion loop buried at an oligomeric contact—a homo-dimer in the flaviviruses (as shown here, with the dimer partner striped for clarity—except that the flavivirus fusion protein spans the membrane twice) or a heterodimer with a companion protein (as in alphaviruses and bunyaviruses). (ii) Triggering of the fusogenic conformational change. The acidic environment of an endosome leads to dimer dissociation and exposure of the fusion loops. (iii) Extended intermediate bridging the two membranes. Multiple monomers at the viral surface insert into the target membrane with concomitant trimerization into an extended intermediate form, separating the two membranes at distances of about 15 nm. The curved arrows indicate the subsequent relocation of domain III to make a hairpin. (iv) Hairpin formation and pulling of the two membranes against each other. Domain III relocation to the side of the trimer redirects the stem toward the fusion loops, thereby pulling the two membranes against each other (gray arrows) to a distance within 1 nm, forcing outer leaflet dehydration to allow membrane contacts. Domain III relocation is accompanied by a domain I internal reorganization, indicated by a red square instead of a red circle. Several adjacent trimers are required to drive fusion (Chao et al., 2014). (v) Fusion pore formation. Upon contact of the two membranes, fusion proceeds via an initial merger of the outer leaflets of the two membranes (“hemifusion”, not shown) followed by formation of a fusion pore, as illustrated here. (B) The HAP2 ectodomain, crystallized in the post-fusion form (corresponding to panels iv and v in A). Left panel: the previously reported X-ray structure of a partially proteolyzed *C. reinhardtii* HAP2 ectodomain to 3.3 Å resolution. The foreground subunit is colored by domains as in (A). The intervening α0 helix is highlighted in brown (brown arrow). Disordered domain II loops are indicated by arrowheads. Black arrows at the bottom end mark the observed limits of the domain I C_0_D_0_ and E_0_F_0_ connections. Middle panel: ribbon representation of the new 2.6-Å resolution structure. The foreground subunit is shown in dark gray with regions not resolved previously highlighted in colors according to the domain they belong to as in (A). A central light blue bar marks the molecular 3-fold axis of the trimer. Right panel: the foreground subunit rotated as indicated and colored by domains to allow labeling the secondary structure elements buried in the trimer. In all panels, disulfide bonds are drawn as green sticks and N-linked glycan chains as sticks colored according to atom type (carbon, white; nitrogen, blue; and oxygen, red). A brown arrow in the middle and right panels marks the region of extended conformation that corresponds to the α0 helix in the left panel. (C) *C. reinhardtii* HAP2 aa sequence on a background colored by domains as in (A) and (B) and secondary structure elements marked. Regions not resolved in the previous structure are boxed. The remaining unstructured regions are on a white background. N-linked glycosylations are marked by full or empty blue stars for resolved sugar residues or disordered glycans, respectively. The gray arrow marks the last residue (592) in the HAP2e expression construct. A dashed box frames the proposed MPER.

We recently reported the X-ray structure of the *C. reinhardtii* HAP2 ectodomain. Although this structure lacked information about important functional regions, it revealed clear homology to class II viral fusion proteins (Fedry et al., 2017), a notion supported by parallel functional studies on HAP2 from other eukaryotic phyla (Angrisano et al., 2017, Pinello et al., 2017, Valansi et al., 2017). The structural study also showed that the HAP2 ectodomain trimerizes upon insertion into membranes, adopting a hairpin arrangement typical of the post-fusion form of the viral proteins, with the transmembrane (TM) proximal region and the fusion loops together at one end of the trimer (Figure 1).

In spite of being structurally unrelated, the three characterized structural classes of viral fusion proteins, termed classes I, II, and III (Igonet and Rey, 2012), were shown to function via a similar mechanism (Harrison, 2008). Anchored in the viral lipid envelope by a C-terminal TM segment, they have a bulky N-terminal ectodomain folded in a metastable conformation at the viral surface. Interactions with the target membrane trigger an exergonic conformational change during which the protein exposes a polypeptide segment, termed “fusion peptide” when it is N-terminal or “fusion loop” when it is internal. This segment inserts firmly into the outer leaflet of the target cell membrane while the protein adopts a transient, elongated trimeric conformation (Figure 1A). The trimer subunits then further re-organize into a hairpin that brings both membranes into close apposition (within 1 nm), thereby overcoming the repulsive force between the two membranes generated by the required dehydration of the outer leaflets to allow lipid contact, helping catalyze the membrane fusion reaction.

The reported HAP2 structure displayed the three characteristic β sheet-rich domains of viral class II fusion proteins, organized around the central domain I. In the post-fusion form, domain I is a 10-stranded β sandwich with the inner A_0_B_0_I_0_H_0_G_0_ β sheet packing against the outer J_0_C_0_D_0_E_0_F_0_ β sheet (Figure 1B). At the domain I “front” end, two of the connections between consecutive β strands make very long excursions to form the elongated domain II: the D_0_E_0_ connection spans domain II β strands *a’* through *e’*, while the H_0_I_0_ connection makes β strands *f* through *l* (Figures 1B and 1C). The fusion loops are located in the segment connecting β strands *c* and *d*, at the very tip of domain II. At the opposite end of domain I—the “bottom” end—a flexible linker connects to domain III, which has an immunoglobulin superfamily fold and is in turn connected to the C-terminal viral TM anchor via a flexible segment called “stem”. Although the structure of HAP2 in the pre-fusion form is not known, structural studies on pre- and post-fusion forms of viral class II fusion proteins (Bressanelli et al., 2004, Gibbons et al., 2004, Guardado-Calvo et al., 2017, Halldorsson et al., 2016, Modis et al., 2004) have shown that the transition mostly involves a rearrangement of the three domains with respect to each other, with domain III undergoing an important translation to re-locate to the sides of the trimer (diagrammed in Figure 1A). Importantly, in all cases studied, the central domain I undergoes the most significant internal rearrangement, with β strand A_0_ switching from the inner to the outer β sheet. This change is accompanied by formation of β strand J_0_, which in the pre-fusion form is part of the linker between domains I and III, and by a significant rearrangement of the A_0_B_0_, C_0_D_0_, and E_0_F_0_ inter-strand connections (Figure 1). These studies suggest that these same regions are also likely to undergo structural changes during the HAP2 trimerization to adopt its post-fusion form.

Although they drive fusion in an overall similar way, the viral fusion proteins of the various classes have different ways of ensuring a stable enough insertion into the target membrane to withstand the molecular gymnastics diagrammed in Figure 1A. In the case of the influenza virus hemagglutinin (HA) (the prototype class I fusion protein), the fusion peptide displays a random coil conformation in the pre-fusion form (Wilson et al., 1981), but restructures upon exposure to a membrane into an α-helical hairpin projecting nine bulky non-polar side chains (Harter et al., 1989, Lorieau et al., 2010). In contrast, for certain class II proteins such as that of the Rift Valley fever virus (RVFV, a bunyavirus), the fusion loop maintains the same conformation it has in the pre-fusion form. In this case, a pre-formed pocket accommodates the head groups of glycerophospholipids of the membrane's outer leaflet by making multiple hydrogen bonds (Guardado-Calvo et al., 2017). Combined with the insertion of only one or two bulky aromatic side chains of the fusion loop, these polar interactions provide stable enough anchoring to drive the fusion process.

Here, we report the X-ray structure of the *C. reinhardtii* HAP2 ectodomain derived from a different crystal form that resolves most of the regions that were missing in the previous structure. It shows that the fusion loops are folded as three amphipathic helices in a horseshoe-shaped arrangement. Mutagenesis altering the non-polar character of the exposed residues in the fusion helices disrupted the ability of the HAP2 ectodomain to insert into liposomes. This structure also showed the presence of an unanticipated cystine ladder-like (CLL) motif reminiscent of the epidermal growth factor (EGF)-like domains inserted in loop C_0_D_0_, packing against a mucin-like region in loop E_0_F_0_ at the bottom end of domain I.

## Results

### Crystallization of the Intact HAP2 Ectodomain

The crystals used to determine the previously reported HAP2 X-ray structure grew only after limited proteolysis of the HAP2 ectodomain (Fedry et al., 2017). Here we screened for crystallization conditions of the unproteolyzed HAP2 ectodomain in complex with HAP2-specific monoclonal antibodies obtained previously (Fedry et al., 2017). The complex with the single-chain variable fragment (scFv) of one of these antibodies resulted in crystals in the hexagonal space group P6_3_22 diffracting to 2.6 Å resolution (Figure S1). Structure determination by molecular replacement using the atomic model of HAP2, PDB: 5MF1, resulted in an electron density map of HAP2 displaying one protomer per asymmetric unit (i.e., with the trimer axis coincident with a crystallographic 3-fold axis) and 80% of the volume occupied by solvent (Table 1). Unexpectedly, this map did not reveal density for a bound scFv. It is possible that formation of the crystal lattice displaced the bound antibody, or that it is present but disordered in the large solvent volumes in between HAP2 molecules (Figure S1). In any event, the electron density allowed tracing the polypeptide chain for most of the HAP2 ectodomain, including loops that were not resolved in the available structure. In total, the new model contains 551 out of 573 residues (Figure 1B), with a break of about 15 residues in the *fg* loop in domain II—a loop projecting laterally, perpendicular to the trimer axis and roughly midway between top and bottom. Potential antibody binding to this loop could explain the absence of scFv density in the crystal, and will be the object of future experiments. Also missing was part of loop E_0_F_0_ at the trimer bottom (Figure S1).

**Table 1:** Data Collection and Refinement Statistics

Wavelength	0.9785
Resolution range	39.0–2.6 (2.7–2.6)
Space group	P6_3_22
Unit cell	
a, b, c (Å)	125.64, 125.64, 367.99
α, β, γ (°)	90, 90, 120
Total reflections	75,352 (1,152)
Unique reflections	37,676 (604)
Multiplicity	2.0 (2.0)
Completeness (%)	70.66 (11.48)
Mean I/sigma(I)	12.32 (1.58)
Wilson B factor	54.09
R_merge_	0.05924 (0.4293)
R_meas_	0.08377 (0.6071)
R_pim_	0.05924 (0.4293)
CC½	0.995 (0.66)
CC^∗^	0.999 (0.892)
Reflections used in refinement	38,050 (604)
Reflections used for R_free_	1,909 (28)
R_work_	0.2244 (0.3323)
R_free_	0.2431 (0.3191)
CC(work)	0.921 (0.743)
CC(free)	0.866 (0.598)
No. of non-hydrogen atoms	4,136
Macromolecules	3,943
Ligands	111
Solvent	82
Protein residues	529
RMSD	
Bond length (Å)	0.010
Bond angle (°)	1.60
Ramachandran plot (%)	
Favored	97.32
Allowed	2.49
Outliers	0.19
Rotamer outliers (%)	1.37
Clashscore	1.75

RMSD, root-mean-square deviation.

Crystallographic refinement led to a final model containing 4,136 atoms in total, with free R factor of 24.3% at 2.6 Å. Comparison of the new model with the previously determined structure resulted in a root-mean-square deviation of 0.53 Å for 528 aligned Cα atoms, indicating that they are very close, with minor changes at the tip of domain II, where the proteolytic treatment to obtain the crystals may have locally affected the protein conformation.

### Potential Protein-Protein Interaction Modules in Domain I

#### Mucin-like Region

**Figure 2:**
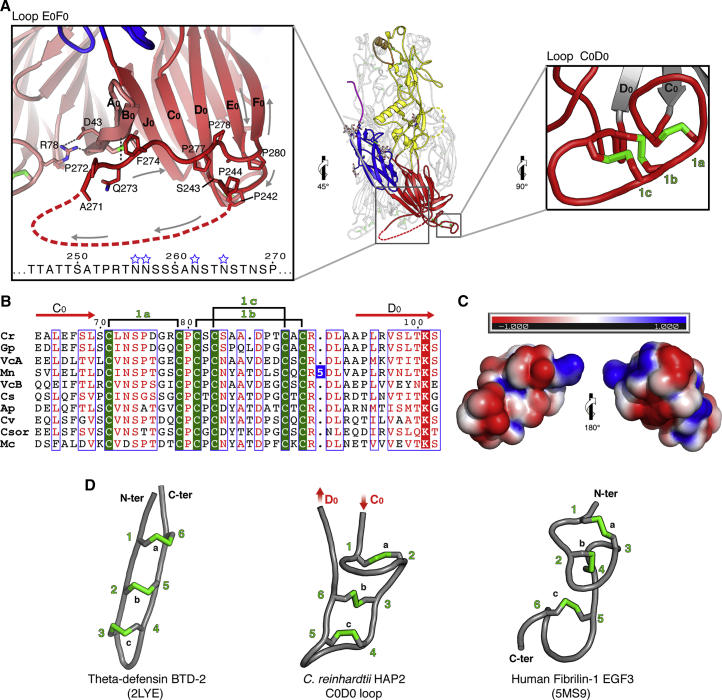
MLR and CLL Motifs in Domain I (A) The HAP2 trimer is shown at the center with boxes indicating the two insets, the E_0_F_0_ (left) and C_0_D_0_ (right) loops. Left panel: the dashed curved line represents the disordered central region of the E_0_F_0_ loop (residues 245–270), with its mucin-like sequence displayed underneath with N-linked glycosylation sequons marked with empty blue stars. Right panel: the CLL motif in the C_0_D_0_ loop with the disulfides drawn in green and labeled as in Figure 1C. (B) Sequence alignment of the CLL motif showing its conservation across green algae, with cysteines in green indicating the disulfide connectivity. One of the sequences has a 5 residue insertion (Blue background). See the STAR Methods for abbreviations. (C) Surface electrostatic potential of the CLL motif displayed on a scale of −1 to 1 kT/e, from red (acidic) through neutral (white) to basic (blue). (D) The C_0_D_0_ loop CLL is shown at the center, next to an authentic cystine ladder motif as observed in θ defensins (Conibear et al., 2012) (left panel) and to an EGF-like module. The cysteines are numbered sequentially to show the different connectivity of the disulfide bonds (green).

The new crystals revealed density for loops C_0_D_0_ and E_0_F_0_ (Figure 2). These two segments are highly variable in sequence and length in HAP2 orthologs across eukaryotes (Figures S2 and S3), and are particularly long in HAP2 from green algae. In *C. reinhardtii* HAP2, the longest is loop E_0_F_0_ (residues 239–284), a 45-residue-long segment rich in proline and serine/threonine residues (Figure S2A), which are typical of heavily O-glycosylated proteins such as the mucins (Shogren et al., 1989) (Figure 2A, left panel, and Figure S2A). Mucin-like regions (MLRs), which are known to confer unique rheological properties on proteins (Tabak, 1995), are present on viral proteins involved in attachment to cells, for instance in glycoprotein C of herpes simplex viruses (Rajcani and Vojvodova, 1998), the respiratory syncytial virus glycoprotein G (Satake et al., 1985, Wertz et al., 1985), or in the surface glycoprotein of Ebola virus (Kirchdoerfer et al., 2017). These viral proteins have in common the recognition and binding of glycosaminoglycans (GAGs) present at the surface of target cells, and the MLR was shown to modulate GAG binding (Altgarde et al., 2015). In animals, MLRs are found on many proteins anchored at the cell surface, as well as in secreted proteins, and were found to modulate cell recognition and adhesion via sugar/lectin interactions (Tran and Ten Hagen, 2013). Although the new crystals did not entirely resolve the E_0_F_0_ loop, they showed density for its N-terminal six amino acids (aa) (sequence PTAPSP, downstream of strand E_0_) as well as its C-terminal 14 residues (271-APQFLSPPAPSTRE-285) preceding β strand F_0_ (Figure 2A), accounting in total for 40% of the loop. The latter proline-rich segment packs against the bottom of domain I, and also contacts loops A_0_B_0_ and C_0_D_0_ of the neighboring subunit. Of note, the downstream F_0_ and G_0_ β strands in the previously reported structure were misassigned and were out of register by two residues, due to poor electron density in this region and the more limited resolution of those crystals. In addition, the density that had been interpreted as a potential O-glycosylation on residue Thr577 at the end of domain III, is clear in the new structure to correspond to an N-linked glycan attached to Asn578. These errors were corrected in the present structure.

#### CLL Motif

The 37-residue long C_0_D_0_ loop is completely resolved in the new structure, and displays a CLL motif with three parallel disulfide bonds (Figure 2A, right panel). This motif is present only in HAP2 from green algae (Figure S3), across which it is highly conserved (Figure 2B). Authentic cystine ladder motifs have been observed in θ defensins (Conibear et al., 2012), which are 18-residue long cyclic peptides with antifungal, antibacterial, and antiviral activities first isolated from primates (Tang et al., 1999). The CLL motif in the C_0_D_0_ loop is however more reminiscent of EGF-like domains (Figure 2D), which are extracellular modules identified in animals, found as single or as multiple copies connected sequentially (Bork et al., 1996). Protein-protein contacts involving EGF-like modules are associated with blood coagulation, complement activation, cell adhesion, or determination of embryonic cell fate during animal development (Bork et al., 1996, Carpenter and Cohen, 1990, Stenflo, 1991). These domains are also around 40 aa long and contain 6 conserved cysteine residues forming three disulfide bonds. The arrangement of these bonds is intermediate between the CLL and the “cystine knot motif” observed in many bioactive peptides (Daly and Craik, 2011). In HAP2, the CLL motif is inserted at the turn of a β hairpin formed by domain I consecutive β strands C_0_ and D_0_, and so the N and C termini are next to each other (Figure 2D, middle panel). In contrast, the “chain of beads” organization of proteins containing sequential EGF-like domains directs their termini to opposite ends (Figure 2D, right panel). This different topology results in a change in the disulfide connectivity, with a 1–3, 2–4, and 5–6 bonding pattern in EGF-like modules and 1–2, 3–6, and 4–5 in the HAP2 CLL (Figure 2B). The HAP2 C_0_D_0_ loop displays an electrostatically charged surface potential (Figure 2C)—a hallmark of EGF-like modules—although the CLL does not display the Ca^2+^ binding motif present in a subset of the EGF-like domains (Rao et al., 1995).

### The Stem Region

**Figure 3:**
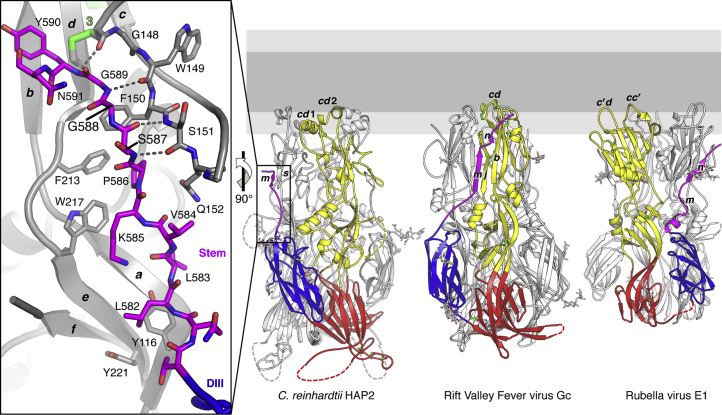
The Stem Inserts into an Adjacent Subunit Domain II Groove HAP2 displayed next to the viral class II glycoproteins for which the stem is resolved: RVFV Gc (PDB: 6EGU) and RV E1 (PDB: 4ADI) in their post-fusion conformation. In HAP2, the stem interacts with domain II of the “left” subunit in the trimer. In RVFV, it interacts with domain II of the same subunit (in yellow). In the RV E1 trimer, both domain III and the stem interact with the subunit to the right. For context, a fused membrane is schematized at the top, with hydrophilic and aliphatic moieties in light and dark gray, respectively. The inset (left panel) shows a more detailed view of the stem (magenta) inserted in the groove formed by the *bc* loop and *aefg* β sheet of the adjacent protomer (gray). Dashed lines represent inter-chain hydrogen bonds between the stem and domain II.

Another region that had not been resolved previously is the stem segment that connects domain III to the C-terminal TM helix. The new structure resolves the N-terminal 12 residues of the stem, showing that it is directed toward the tip of domain II of the adjacent “left” subunit in the trimer (Figure 3). Compared with the two viral class II proteins for which the stem is resolved, RVFV Gc and rubella virus (RV) E1, the path of the stem to reach the fusion loops in the HAP2 post-fusion trimer is different. In spite of a similar positioning of domain III, the stem does not turn back to interact with domain II from the same protomer (yellow in Figure 3) as in RVFV. HAP2 is also different to RV E1, as in this case not only the stem but also domain III interacts with the alternative adjacent subunit (to the “right” in the trimer; Figure 3, right panel). The HAP2 stem fills a surface groove in the adjacent protomer, formed between the *bc* loop and the *aefg* β sheet of domain II. The polar contacts include a short segment of antiparallel main-chain β interactions (strand *m,* residues 587–589; Figures 1B and 1C) with the *bc* loop, inducing formation of strand *s* (residues 149–151; Figure 3, inset). The construct used for crystallization ended at residue G592, followed by the affinity tag. The last residue ordered in the structure is N591 (Figure 1C), with the stem ending roughly at the level of the *bdc* β sheet in the domain II tip. In full-length *C. reinhardtii* HAP2, there are 42 aa between this point and the TM helix, including an amphipathic membrane proximal external region (MPER). Comparison with the viral proteins (Figure 3) suggests that the distance remaining to reach the fusion loops would be spanned by about 10–12 residues, until about position 604. The sequence of the remaining C-terminal part (residues 605–633, Figure S4) is compatible with the MPER present in a number of viral fusion proteins (Cai et al., 2011, Guardado-Calvo and Rey, 2017, Shogren et al., 1989). The MPER has so far eluded structural characterization for any fusion protein in the context of the post-fusion form (the MPER is not represented, for clarity, in the diagram of Figure 1A).

### The HAP2 Membrane Insertion Region

**Figure 4:**
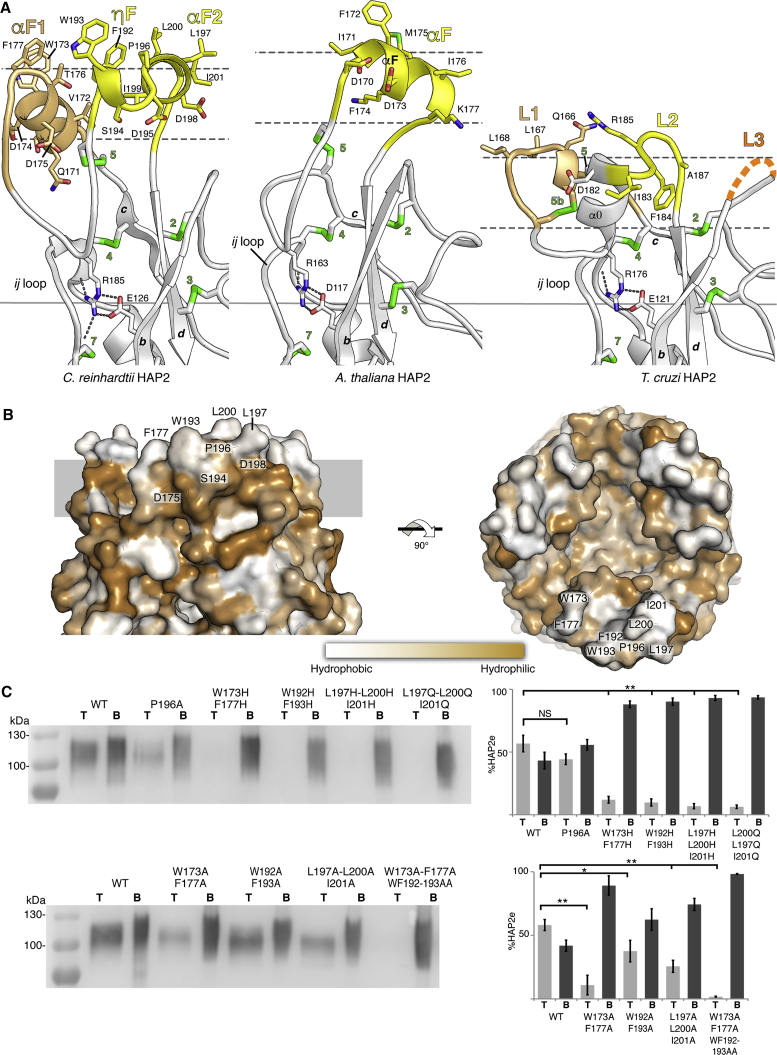
The *C. reinhardtii* HAP2 Membrane Insertion Surface (A) The tip of *C. reinhardtii* HAP2 domain II (left) compared with the corresponding region in *A. thaliana* (center) and *T. cruzi* (right) (Fedry et al., 2018). The helices in fusion loops 1 and 2 are colored beige and yellow. Broken orange lines mark a two-residue disordered segment in the *bc* loop in *T. cruzi* HAP2 that could potentially also insert into the target membrane. The panels were aligned on the conserved salt bridge (indicated by a continuous horizontal line), showing that the membrane would be at a variable distance from the *bdc* core β sheet (the horizontal dashed lines mark roughly the limits of the polar moiety of the membrane's outer leaflet). This figure highlights the potential malleability of the membrane insertion surface. (B) Surface representation of the HAP2 trimer tip colored by hydrophobicity. The gray stripe indicates a roughly polar head group layer of the membrane. Note the space left at the center by the projecting domain II tips of the three trimer subunits, seen on the right panel by depth cueing with white fog. (C) Membrane binding analysis of *C. reinhardtii* wild-type (WT) and fusion helix mutants of HAP2 by liposome co-flotation assays on iodixanol gradients (Optiprep). Top (T) and bottom (B) fractions of the gradients were analyzed by western blotting for the presence of the HAP2 ectodomain. The right panels show the quantification of HAP2e detected in the gradient fractions. The values represent the average of three independent experiments ± SD, analyzed by Student's t test. Levels of significance are: ^∗^p < 0.05; ^∗∗^p < 0.01; NS, not significant.

**Figure 5:**
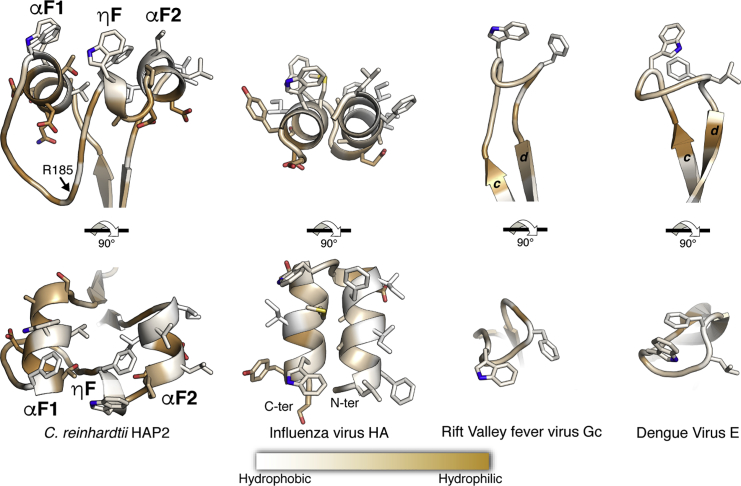
Comparison of the *C. reinhardtii* HAP2 fusion surface with viral counterparts The HAP2 fusion loops (left panel) form a platform projecting hydrophobic residues. As similar platform is formed by the fusion peptide of the class I influenza virus hemagglutinin (PDB: 2KXA) (second panel). The fusion loop of the RVFV (PDB: 6EGU) or the flavivirus fusion protein (PDB: 1OK8) (right panels) is significantly less extensive.

The packing between HAP2 trimers in the new crystals involves 2-fold symmetric head-to-head interactions along the 3-fold crystallographic axis (Figure S1). This “dimer of trimers” is likely to correspond to the “hexamer” observed in the size-exclusion chromatography (SEC) profile of the purified recombinant HAP2 ectodomain (Figure S1A, left panel). In this assembly, the non-polar residues at the tip of domain II are masked from solvent. This interaction between trimers results in the fusion loops being entirely resolved in the new structure. The segment between β strands *c* and *d* of domain II, which is 40 residues long, projects two loops (*cd1* and *cd2* in Figure 3B) exposing non-polar side chains. In place of the single αF fusion helix observed in *A. thaliana* HAP2 and the three potential short fusion loops of *T. cruzi* HAP2 (Figure 4A), in *C. reinhardtii* the HAP2 membrane insertion surface is composed of helix αF1 in loop *cd1* and αF2 in *cd2*, running roughly antiparallel to each other (Figure 5, left panels), and a small 3_10_ helix (ηF) also in loop *cd2* directly preceding αF2. The three “fusion helices” are arranged into a horseshoe shape, exposing the non-polar residues toward the target membrane (Figures 4 and 5). αF1 is 9 aa long and exposes residues W173 and F177; ηF is only one turn and exposes F192 and W193, and αF2 is 7 aa long and exposes P196, L197, L200, and I201 (Figure 4). In addition to providing a firm hold of the protein onto the target membrane, the substantial membrane insertion surface is likely to generate a significant stress upon insertion into the outer leaflet of the opposing gamete membrane. The resulting lipid bilayer destabilization can potentially also play a role in facilitating the downstream membrane fusion process.

The HAP2 *cd* segment is long in comparison with the *cd* loop of the fusion protein of the well-studied flaviviruses (10 residues) (Modis et al., 2004) or bunyaviruses such as RVFV (15 residues) (Guardado-Calvo et al., 2017), but not when compared with other class II viral fusion proteins. For instance, RV E1 has a 48-aa-long *cd* inter-strand connection (DuBois et al., 2013), featuring an additional β strand (*c’*) that augments the *bdc* β sheet at the domain II tip into *bdcc’* and exposes two fusion loops, *cc’* and *c'd* (Figure 3B). In HAP2, *cd1* and *cd2* are separated by the arginine residue (Figure 4A) making a strictly conserved salt bridge (R185-E126 in *C. reinhardtii* HAP2) with a glutamic acid in β strand *b* (Fedry et al., 2017). This salt bridge is a distinctive HAP2 feature among all class II proteins, linking the variable membrane insertion region to the highly conserved core of the protein (Fedry et al., 2018). The R185 side chain not only makes a salt bridge but also donates multiple hydrogen bonds to backbone carbonyls of the highly conserved “*ij* loop” (Figure 4A), so named because it takes the place of the *ij* β hairpin of viral class II proteins. The HAP2 *ij* loop is the central part of the pfam 10699 motif (Finn et al., 2014), a ∼50-aa-long stretch of conserved sequence that allowed the identification of HAP2 in numerous eukaryotic species.

Although the aa 184–190 stretch (i.e., including the conserved R185) in between *cd1* and *cd2* was ordered in the previous structure and formed an α helix termed α0, it displays an extended conformation in the new structure (Figures 1B and 4A, left panel). This segment is also extended in *Arabidopsis thaliana* HAP2 (Figure 4A, middle panel) (Fedry et al., 2018). The difference between the two *C. reinhardtii* HAP2 structures in this stretch could have been induced by the *in situ* proteolytic treatment used to grow the initial crystals. α0 helix folding/unfolding could also be a mechanism required to fully project the *cd* loop toward the target membrane during the fusogenic conformational change of HAP2. Indeed, the crystal structure of domain II of *T. cruzi* HAP2 does display an α0 helix in the same location and orientation (Figure 4A, right panel). In contrast to the fusion helices, α0 runs perpendicular to the target membrane. The only non-polar bulky residues present at the membrane insertion region are in the second turn of the *T. cruzi* HAP2 α0 helix, and are not fully exposed (Figure 4A, third panel), suggesting that this segment may have adopted an alternative conformation in the aqueous environment used for crystallization (Fedry et al., 2018).

### The Non-polar Residues of the Fusion Helices Are Required for Liposome Binding

Our previous results demonstrated that gamete fusion *in vivo* correlates with the ability of HAP2 to insert into liposomes *in vitro* (Fedry et al., 2017, Fedry et al., 2018). To confirm that the exposed residues of the fusion helices are indeed important for membrane insertion, we examined the behavior of the HAP2 ectodomain and of selected mutants in liposome co-flotation in density gradients, as described in the STAR Methods section. All the mutants tested were similar to wild-type in terms of expression yields and transit through the quality control system of the producer cells for secretion. The SEC profiles of the mutants were also similar to wild-type, indicating that the introduced mutations did not induce any obvious misfolding, as expected for exposed residues such as those being tested.

As P196 induces an important kink causing the shift from ηF to αF2 in loop *cd2*, we tested the effect of the P196A mutation and found that there were no statistically significant differences with wild-type (Figure 4C) in liposome co-flotation. The kink induced by P196 in the conformation of the fusion helices is thus not essential for insertion into membranes. In contrast, we found a more drastic effect when we substituted the non-polar residues by histidine, a polar aa. Indeed, the double mutants W173H/F177H (αF1), W192H/F193H (ηF), and the triple mutant L197H/L200H/I201H (αF2), displayed essentially no detectable liposome binding. The triple mutant L197Q/L200Q/I201Q had the same effect, suggesting that any polar side chain replacing the non-polar bulky side chains of these helices will interfere with membrane binding, as expected. This last result is also an important control, as the introduction of multiple neighboring histidines could induce local repulsion upon their protonation, although the membrane binding assays were done at pH 7.4 and the pK_a_ of solvent-exposed histidine side chains is 6.8. Of note, in contrast to virus fusion driven by class II fusion proteins, which takes place in the acidic environment of the endosomes, *Chlamydomonas* gamete fusion occurs in the extracellular environment at neutral pH.

When the same bulky non-polar residues were replaced by alanine, which has a small non-polar side chain, the effect on membrane insertion was less striking: the difference in binding of double mutant W192A/F193A in ηF was less significant with respect to wild-type, and W173A/F177A (αF1) or L197A/L200A/I201A (αF2) displayed reduced binding although not as significant as the mutants with polar residues. It required a mutant with alanine substitution in both, αF1 and ηF, W176A/F177A/W192A/F193A, for a complete loss of binding comparable with the experiments involving substitution by polar residues (Figure 4C). These experiments therefore demonstrate that the bulky non-polar residues of the fusion loops indeed control the insertion of the HAP2 ectodomain into lipid bilayers. The differences observed when substituting them to alanine instead of polar residues suggest that certain positions are key anchoring sites requiring a bulky non-polar side chain, whereas others are necessary only to maintain the non-polar character of the surface. This is demonstrated in particular by the significant difference in insertion to liposomes of the mutant W173A/F177A compared with W192A/F193A (Figure 4C, bottom panels).

## Discussion

The X-ray structure of the *C. reinhardtii* HAP2 ectodomain described here has higher resolution and provides views of important functional regions that were not resolved in the previously reported structure. One crucial segment that was missing is the tip of domain II and its fusion loops, which must insert into the target membrane and are critical for the membrane fusion function of HAP2. The new structure shows that the domain II tip exposes a hydrophobic platform formed by three short helices projecting ten bulky non-polar residues that are used for membrane insertion (Figure 1A, steps ii to v). This region is involved in hydrophobic head-to-head interactions between two HAP2 trimers to form the crystals (Figure S1B), making a dimer of trimers that was also observed in solution (Figure S1A, left panel).

The arrangement of the membrane insertion surface in *C. reinhardtii* HAP2 is reminiscent of the α-helical hairpin of the influenza virus HA fusion peptide, which projects nine bulky non-polar residues (Figure 5, second panel) into the target membrane (reviewed in Apellaniz et al., 2014). This structure forms only upon interaction of the HA fusion peptide with the outer leaflet of the membrane, whereas in HAP2 the helical arrangement appears to be pre-formed in the protein. An important difference is that in HA the two antiparallel amphipathic helices pack very tightly against each other, whereas in HAP2 they have a rather loose, horseshoe-shaped arrangement. The HAP2 fusion loops are also very different to those of the viral class II proteins that have been characterized, such as the fusion proteins of flaviviruses—i.e., dengue virus E (Klein et al., 2013, Modis et al., 2004), of Gc from bunyaviruses such as RVFV (Guardado-Calvo et al., 2017) (Figure 5, right panels), and also to the RV E1 fusion loops (Figure 3B). Molecular dynamics analyses using RVFV Gc showed that insertion of the fusion loop involves a rearrangement of the membrane such that the bulky lipid head groups move apart to avoid clashes with the main chain of the inserted loop. In addition, the presence in the membrane of lipids with very small polar heads was required to fill the void created in the aliphatic moiety at the site of insertion. This effect would be more drastic in the case of *C. reinhardtii* HAP2 as the inserted motif is much larger (compare panels in Figures 4A and 5). We note that the tips of the three domains II in the HAP2 trimer leave an empty space at the center (Figure 4B), which we propose is important to accommodate the displaced bulky lipid head groups upon insertion of the fusion helices. In influenza virus HA2, the tight α-helical platform of the fusion peptide is loosely tethered via a flexible linker to the central, rigid trimeric α-helical coiled coil of the HA2 subunit of HA (Bullough et al., 1994, Chen et al., 1999), providing flexibility and leaving space to accommodate the displaced lipid head groups. In HAP2, not only the lateral packing of the fusion helices appears to be loose (the minor effect of the P196A mutation in liposome binding shown in Figure 4C indeed suggest that this region displays conformational malleability), but their connection to the rigid *bdc* β sheet of the domain II tip also displays flexibility (Figure 4A) that is potentially important for membrane insertion. The relatively flexible connection to the *bdc* β sheet could combine with a potential rocking about the strictly conserved R185-E125 salt bridge (Figure 4A). The malleability of this region is further exemplified by formation or not of a vertical α0 helix around the R185 position. We also note that sequence analysis of the HAP2 fusion loop region further suggests that the three fusion helices are not conserved in many algae (Figure S5), and some appear to have a single fusion helix, similar to HAP2 from the flowering plants (Fedry et al., 2018). Future studies using molecular dynamics simulations and taking advantage of the currently available X-ray structures of three orthologs should be useful to better understand the HAP2 membrane insertion process.

A driver for the high variability observed in certain regions of HAP2 may be the requirement of barriers that avoid cross-species fertilization. In the viral class II proteins, the fusion loops are exposed only at the time of fusion, and until this moment the protein is maintained in a metastable conformation via interaction with itself (as diagrammed in Figure 1A, panel i) or most commonly with other proteins, such that that the fusion loops are masked (Guardado-Calvo and Rey, 2017, Kielian, 2006). The high sequence variability of the HAP2 fusion loops could thus be driven by its necessary interaction with species-specific proteins that recognize and bind to this region. The controlled release of their grip on HAP2 could then be a way to trigger its fusogenic conformational change and exposure of the fusion helices (corresponding to panel ii in Figure 1A). In our *in vitro* experiments, in the absence of the putative interacting molecules normally present in the *in vivo* situation, the recombinant HAP2 ectodomain is triggered spontaneously to change conformation, with monomers trimerizing irreversibly in solution to form post-fusion trimers that interact head-to-head (Figure S1). The remaining monomers in our preparations remain competent for membrane insertion, by forming membrane-inserted post-fusion trimers when incubated with liposomes (Fedry et al., 2017, Fedry et al., 2018). Cellular fusion events are normally controlled such that they can take place fast but only at the required time and space. This is demonstrated, for instance, in neurotransmitter release, where the fusogenic SNARE proteins are controlled by an array of other synaptic proteins that allow for very fast fusion of vesicles that are pre-bound at the cytosolic side of the plasma membrane. The rate-limiting steps involved in priming the fusion machinery have already taken place when a signal provided by calcium influx activates fusion (Brunger et al., 2018). In the case of HAP2-driven gamete fusion, our results now suggest that, in addition to fusion loop masking, there may be additional controls at other steps—such as trimerization, which involves rearrangements in the bottom end of domain I. Our data further suggest additional potential means of identifying interaction partners of HAP2 *in vivo*, for instance by comparative co-immunoprecipitation using HAP2 wild-type and variants mutated at key sites at the domain I bottom.

## STAR★Methods

### Key Resources Table

**Table:** 

REAGENT or RESOURCE	SOURCE	IDENTIFIER
**Antibodies**		

A20	This study	RRID: AB_2749850

**Deposited Data**		

*C. reinhardtii* HAP2 ectodomain structure	This study	PDB: 6E18
*C. reinhardtii* HAP2 *in situ* proteolyzed ectodomain	Fedry et al., 2017	PDB: 5MF1

**Experimental Models: Cell Lines**		

*D. melanogaster* S2 cell line	Thermo-Fisher	Cat # R690-07
Expi293F cells	Thermo-Fisher	A14635

**Recombinant DNA**		

pMT/BiP/TwinStrep		N/A
pcDNA3.1(+)/CD5sp/TwinStrep		N/A

**Software and Algorithms**		

XDS	Kabsch, 1988	http://xds.mpimf-heidelberg.mpg.de/
CCP4	Collaborative Computational Project, 1994	http://www.ccp4.ac.uk/
Phaser	McCoy et al., 2007	http://www.phaser.cimr.cam.ac.uk/index.php/Phaser_Crystallographic_Software
Coot	Emsley et al., 2010	https://www2.mrc-lmb.cam.ac.uk/personal/pemsley/coot/
AutoBuster	Bricogne et al., 2010	https://www.globalphasing.com
Staraniso	Global Phasing Limited	http://staraniso.globalphasing.org/cgi-bin/staraniso.cgi
MolProbity	Chen et al., 2010	http://molprobity.biochem.duke.edu/
Pymol	Molecular Graphics System, Schrodinger, LLC	https://pymol.org/2/
Heliquest	Gautier et al., 2008	http://heliquest.ipmc.cnrs.fr/
ImageJ	Schneider et al., 2012	https://imagej.nih.gov/ij/

**Other**		

*Chlamydomonas reinhardtii*	Uniprot: A4GRC6	N/A
*Gonium pectoral*	Uniprot: X5IBH0	N/A
*Volvox carteri* form A	Uniprot: D8U2H4	N/A
*Monoraphidium neglectum*	Uniprot: A0A0D2MSE3	N/A
*Volvox carteri* form B	Uniprot: D8U2H3	N/A
*Coccomyxa subellipsoidea*	Uniprot: I0Z782	N/A
*Chlorella variabilis*	Uniprot: E1Z455	N/A
*Chlorella sorokiniana*	Uniprot: A0A2P6TVX6	N/A
*Micractinium conductrix*	Uniprot: A0A2P6VMS5	N/A
*Auxenochlorella protothecoides*	Uniprot: A0A1D2A410	N/A
*Cyanidioschyzon merolae*	GenBank: XP_005536505.1	N/A
*Selaginella moellendorfii*	GenBank: XP_002971451.1	N/A
*Arabidopsis thaliana*	Uniprot: AAY51999.1	N/A
*Capsella rubella*	GenBank: XP_006289435.1	N/A
*Erythranthe guttata*	GenBank: XP_012846387.1	N/A
*Solanum lycopersicum*	GenBank: XP_019070618.1	N/A
*Lilium longiflorum*	Uniprot: BAE71142.1	N/A
*Zea mays*	GenBank: XP_020531486.1	N/A
*Oryza sativa*	Uniprot: BAF16968.1	N/A
*Amborella trichopoda*	GenBank: XP_020531486.1	N/A
*Toxoplasma gondii*	UniProt: KYF43852.1	N/A
*Plasmodium falciparum*	GenBank: XP_001347424.1	N/A
*Eimeria tenella*	Uniprot: BAM16295.1	N/A
*Tetrahymena thermophila*	Uniprot: AIA57699.1	N/A
*Paramecium tetraurelia*	Uniprot: CAK65033.1	N/A
*Trypanosoma cruzi*	Uniprot: EAN93043.1	N/A
*Trypanosoma congolense*	Uniprot: CCC94129.1	N/A
*Trypanosoma brucei*	Uniprot: EAN78468.1	N/A
*Leishmania major*	GenBank: XP_003722443.1	N/A
*Dictyostelium discoideum*	Uniprot: BAS29571.1	N/A
*Physarum polycephalum*	Uniprot: BAE71144.1	N/A
*Monosiga brevicolis*	Uniprot: EDQ88884.1	N/A
*Nematostella vectensis*	Uniprot: EDO36432.1	N/A
*Hydra vulgaris*	GenBank: XP_004211319.2	N/A
*Capitella teleta*	Uniprot: ELU07639.1	N/A
*Tribolium castaneum*	Uniprot: EFA06462.1	N/A
*Acyrthosiphon pisum*	GenBank: XP_016661643.1	N/A
*Orchesella cincta*	Uniprot: ODN05384.1	N/A
*Saccoglossus kowalevskii*	GenBank: XP_006821859.1	N/A

### Contact for Reagent and Resource Sharing

Further information and requests for reagents may be directed to and will be fulfilled by the Lead Contact, Félix A. Rey (felix.rey@pasteur.fr).

### Experimental Model and Subject Details

#### Insect Cell Culture

Drosophila S2 cells were maintained in flasks at 28°C in HyClone medium (GE Healthcare Life Sciences) supplemented with 50 units/mL of penicillin, 50 units/mL of streptomycin and 7 μg/mL of puromycin for selection of stable transfectans. Large-scale cultures for protein production were grown in spinner flasks at 100 rpm. No information on the sex of these cells is available.

#### Mammalian Cell Culture

Expi293F cells were grown at 37°C in Expi293 expression medium (Thermo Fisher) with rotary agitation at 135 rpm. No information on the sex of this cell line is available.

### Method Details

#### Expression Construct for the Single Chain Variable Fragment (scFv) of Monoclonal Antibody A20

Monoclonal antibodies targeting *C. reinhardtii* HAP2 were generated as described previously (Fedry et al., 2017). For sequencing of the antibody variable regions total RNA was prepared from A20 hybridoma cells and cDNA was synthesized using separate constant region primers for heavy and light chain. Variable regions of heavy and light chain genes were subsequently amplified using gene-specific primers and subcloned into a plasmid for insect cell expression of scFvs described previously (Gilmartin et al., 2012).

#### Protein Expression and Purification

*Drosophila* S2 stable cell lines expressing soluble C-terminally truncated *Chlamydomonas reinhardtii* HAP2 ectodomain (residues 23-592), were generated to obtain high yields of recombinant proteins for crystallization experiments (Fedry et al., 2017). The expression construct included an enterokinase cleavage site (sequence DDDDK) immediately downstream HAP2 G592. at the C-terminal end of the ectodomain, followed by a double strep-tag for purification. The expression of the protein is under the control of metallothionein promoter, which is induced by adding 4 μM CdCl2 to the cell culture medium when the cell density reaches approximately 7x10^6^ cells/ml. At 5 days post-induction, the cells were pelleted and the soluble ectodomain was purified by affinity chromatography from the supernatant on a *StrepTactin Superflow* column. The strep tag was then removed by treatment with enterokinase and the protein was further purified by SEC on a *Superdex200* column equilibrated with 10 mM bicine pH 9.3. As we reported previously (Fedry et al., 2017), low ionic strength conditions at pH 9.3 reduce formation of HAP2 hexamers. As shown in Figure S1, hexamers appear to form by head-to-head interaction between two trimers via the hydrophobic fusion loops, thus interfering with HAP2 binding to membranes.

The yields of HAP2 ectodomain obtained from S2 cells were around 15 mg/l of cell culture. The purified protein was concentrated to approximately 5 mg/ml.

We also generated an S2 stable cell line expressing antibody A20 scFv, which was expressed following the same procedures used for the HAP2 ectodomain. The complex between the HAP2 ectdomain and scFv A20 was obtained mixing both proteins in a molar ratio 1:2 and incubating overnight at 4°C. The HAP2-scFv A20 complex was then separated from unbound scFv on a Superdex200 column. The fractions in Figure S1, right panel, corresponding to the complex were pooled and then concentrated to approximately 7 mg/ml.

#### Crystallization and Structure Determination

Crystals were grown using the sitting drop method at 293K by mixing HAP2-scFv A20 protein solution in a ratio 1:1 with reservoir solution containing 0.2M LiSO_4_, 0.1M Tris-HCl pH 8.5 and 25% w/v PEG 5000 MME. Small hexagonal plates obtained after 5 days were cryoprotected with reservoir solution containing 15% glycerol and plunged in liquid nitrogen.

Data collection was carried out at the SOLEIL synchrotron (St Aubin, France). Data were processed, scaled and reduced with XDS (Kabsch, 1988) and AIMLESS (Evans and Murshudov, 2013). Diffraction anisotropy was corrected with DEBYE and STARANISO programs using the STARANISO server (http://staraniso.globalphasing.org/). These programs perform an anisotropic cut-off of merged intensity data, a Bayesian estimation of structure amplitudes and apply an anisotropic correction to the data. The HAP2 structure was determined by molecular replacement using PHASER and the coordinates of the previous crHAP2e ectodomain (PDB accession code 5MF1) as search model. The model was corrected and completed using COOT (Emsley et al., 2010) and the corrected anisotropic amplitudes were used for model refinement in BUSTER (Bricogne et al., 2010). The stereochemical properties of the model were validated with MolProbity (Chen et al., 2010).

#### Design and Expression of HAP2 Fusion Loops Mutants

Recombinant HAP2 ectodomain and fusion loops mutants for liposome co-flotation experiments were obtained by transient transfection of mammalian cells, by using the pcDNA3.1(+) plasmid with the gene coding for the HAP2 ectodomain (residues 23-592) followed by a double strep-tag at the C-terminal end of the ectodomain. Mutants at positions 173, 177, 192, 193, 197, 200 and 201 were generated by standard PCR methods. Expi293 cells were transfected with WT or mutant constructs using polyethylenimine (PEI, 1 mg/ml) in a DNA:PEI ratio 1:4. Cell culture supernatants were harvested at 5 days post-transfection and protein was purified by affinity chromatography as mentioned above. As for the protein produced in S2 cells, the purified proteins were buffer exchanged to 10 mM bicine pH 9.3 and concentrated to 1 mg/ml, to minimize hexamer formation.

The yields of HAP2 WT expressed in mammalian cells were around 200 μg of pure protein per 30 ml of cell culture. The yields of all HAP2 fusion loop mutants tested were in the range of 1 to 2 times the yields of the WT protein.

#### Liposome Co-flotation Experiments

Liposomes composed of DOPE (1,2-dioleoyl-sn-glycero-3-phosphoethanolamine), DOPC(1,2-dioleoyl- sn-glycero-3-phosphocholine), cholesterol and sphingomyelin in a molar ratio1/1/3/1, were prepared by the freeze-thaw and extrusion method. 1μM protein was mixed with1 mM freshly prepared liposomes and incubated for 1h at 25°C in 100 mL PBS. Samples were adjusted to a final concentration of 40% Optiprep™ (in PBS), loaded in centrifuge tubes and overlaid with 4.5 mL 20% Optiprep™ and 0.3 mL PBS. Centrifugations were performed overnight at 4°C and 192000 x g on SW55ti rotor. Top and bottom fractions of the gradient were analyzed by immunoblotting using anti-Strep tag antibody.

#### Illustrations

Cartoon representations were made with PyMol (Schrodinger, 2015.). The HAP2 amino acid sequence alignments displayed were made by MUSCLE (Edgar, 2004) and rendered with ESPript 3.0 (Robert and Gouet, 2014). The organisms represented in Figure 2 have the following abreviations: *Chlamydomonas reinhardtii* (Cr), *Gonium pectoral* (Gp), *Volvox carterii* (forms A and B; VcA and VcB respectively), *Monoraphidium neglectum* (Mn), *Coccomyxa subellipsoidea*, (Cs), *Auxenochlorella protothecoides* (Ap), *Chlorella variabilis* (Cv), *Chlorella sorokiniana* (Csor) and *Micractinium conductrix* (Mc).

### Quantification and Statistical Analysis

Densitometry analysis of western blots from liposome co-flotation assays was performed with ImageJ software (Schneider et al., 2012) and expressed as percentage of HAP2e in top and bottom fractions of iodixanol gradients. Results are shown as mean ± SD of three independent experiments (n=3). Differences in liposome binding between HAP2e WT and mutants were analyzed by a Student’s t-test (P<0.05, ^∗^; P<0.01, ^∗∗^).

### Data and Software Availability

The accession number for the *Chlamydomonas reinhardtii* HAP2 structure reported in this paper is PDB: 6E18.

## Supplemental Information

Document S1. Figures S1–S5

Document S2. Article plus Supplemental Information
