# Supplementary material for: Species-Specific Functional Regions of the Green Alga Gamete Fusion Protein HAP2 Revealed by Structural Studies
Source: Structure. 2019 Jan 2;27(1):113–124.e4. doi: 10.1016/j.str.2018.09.014 (PMC6327110; doi:10.1016/j.str.2018.09.014)

Structure, Volume *27*

Supplemental Information

Species-Specific Functional Regions of the Green Alga Gamete Fusion Protein HAP2 Revealed by Structural Studies

Eduard Baquero, Juliette Fedry, Pierre Legrand, Thomas Krey, and Felix A. Rey

Supplemental Information for manuscript:

Species-specific functional regions of the green alga gamete fusion protein HAP2 revealed by structural studies

Eduard Baquero[1,2], Juliette Fedry[1,2,#], Pierre Legrand[3], Thomas Krey[1,2,#] and Felix A. Rey[1,2,¶]

[1]Institut Pasteur, Unité de Virologie Structurale, 25-28 Rue du Docteur Roux, 75724 Paris Cedex 15, France
[2]CNRS UMR 3569, 25-28 Rue du Docteur Roux, 75724 Paris Cedex 15, France
[3]Synchrotron SOLEIL, L'Orme des Merisiers, 91192 Gif-sur-Yvette, France
[¶]Lead contact.

[#]Present address: Cryo-Electron Microscopy, Bijvoet Center for Biomolecular Research, Utrecht University, 3584 CH Utrecht, the Netherlands (JF) ; Institute of Virology, Hannover Medical School and German Center for Infection Research, 30625 Hannover Germany (TK)

Contents :

Legends to Supplemental Figures S1-S5

Supplemental Figures S1-S5

Figure S1 (related to Figure 1). HAP2 SEC elution profiles and crystal packing. A) The left panel shows the elution profile of purified recombinant HAP2 ectodomain, showing a peak that corresponds to a monomer (peak marked "M"), which irreversibly converts to a high-molecular weight form consistent with a HAP2 hexamer (peak marked H). The right panel shows the elution of the protein from the "M" peak in the left, when mixed with an excess of scFv A20 (see STAR Methods). Addition of the scFv appears to drive formation of a complex eluting as a trimer. SDS PAGE analysis of this peak (inset) stained with Coomasie blue shows that it contains both HAP2 and scFv A20.

B) Packing of HAP2 molecules in the crystals obtained with protein from the fractions corresponding to the HAP2/scFv A20 complex (left peak in A, right panel). The packing shows two trimers of the HAP2 ectodomain interacting head-to head, masking the HAP2 membrane insertion region from solvent (indicated by small horizontal black arrows in the right panel). In turn, these dimers of trimers further assemble via lateral interactions of the domains I and III moieties, to make a crystal belonging to space group $P6_322$. The left panel shows a view down the hexagonal helicoidal axis of the crystal, and the right panel a view down an orthogonal 2-fold axis. The red arrows in the right panel point to the region occupied by the disordered part of loop E_0F_0 in domain I, and the orange arrows mark the location of the disordered loop *fg* in domain II. The space occupied by solvent in the crystal is big enough to allow the presence of the scFv A20, if bound to one of the disordered regions of HAP2. The inset below the right panel is a close-up of the lateral packing between trimers in the crystal, which involves packing of the CLL motif of loop C_0D_0 against domain III of an adjacent trimer, and also involves sugar residues as well as the partially disordered mucin-like region.

C) Elution profile of the wild type HAP2 ectodomain expressed in mammalian Expi293 cells (black curve) together with a representative fusion helix mutant (grey curve). These proteins were used for the experiments of Figure 4C. Note that expression in lower amounts of these constructs (which were not conceived for structural studies) in mammalian cells results in a shoulder in the SEC profile corresponding to a HAP2 trimer, in addition to the hexameric form. The amount of trimer seems to be higher for the mutant, in line with the reduced hydrophobicity of its fusion surface.

Figure S2 (related to Figure 2). Amino acid sequences in the HAP2 E_0F_0 loops (A) from green algae and (B) other taxa. Similar amino acids across different taxa are showin red and framed in blue. A grey background in the *C. reinhardtii* sequence indicates the residues of the

E_0F_0 that were disordered in the new HAP2 crystals. Potential N-linked glycosylation sites in the loop are marked by blue empty stars (as in Figure 1C).

Figure S3 (related to Figure 2). Amino acid sequences in the HAP2 C_0D_0 loops. Similarity across taxa is displayed as in figure S2. The conserved cysteines in the C_0D_0 loop of HAP2 from green algae are shown in a green background.

Figure S4 (related to Figure 3). Amino acid sequences in the stem region of HAP2. Amino acid similarity across taxa is displayed as in Figure S2. The conserved cysteines are highlighted in green. A blue background indicates amino acid insertions with respect to the *C. reinhardtii* HAP2 sequence. A linear diagram of this HAP2 region is drawn above the sequence: blue marks domain III, magenta the stem and the MPER, and grey the TM segment. A black box marks the segment resolved in the new structure. Note the different pattern of cysteines in this region in HAP2 from distant protozoa and from animals. Blue stars mark glycosylation sites, as in Figure 1C.

Figure S5 (related to Figure 4). Variability of the fusion loops across green algae.
(A) The amino acid sequence alignment of HAP2 from green algae displayed in the region of the *bcd* β-sheet. A red background indicates conserved residues. Highlighted in yellow are segments corresponding to the helical regions of fusion loops 1 and 2 displayed with Heliquest (Gautier et al., 2008) in panel B. A blue background denotes residue insertions as in Figure S4. Disulfide bridges are indicated with green numbers, and the residues making the conserved salt bridge are marked by a vertical red arrow. Note that a number of algae have a deletion in the region of αF1, and in this respect appear more similar to HAP2 from flowering plants (see Figure 4A).
(B) Amphipathic helices were predicted using the Heliquest server. The size of the circles is proportional to the amino acid side chain volume. Positively charged residues are colored red and negatively charged residues are in blue. Glycines are shown in gray and polar uncharged residues are colored pink and purple. Hydrophobic residues are displayed in yellow. Prolines are shown in green. The hydrophobic moment value and its vector are indicated for each helix.

Supplemental Figure S1

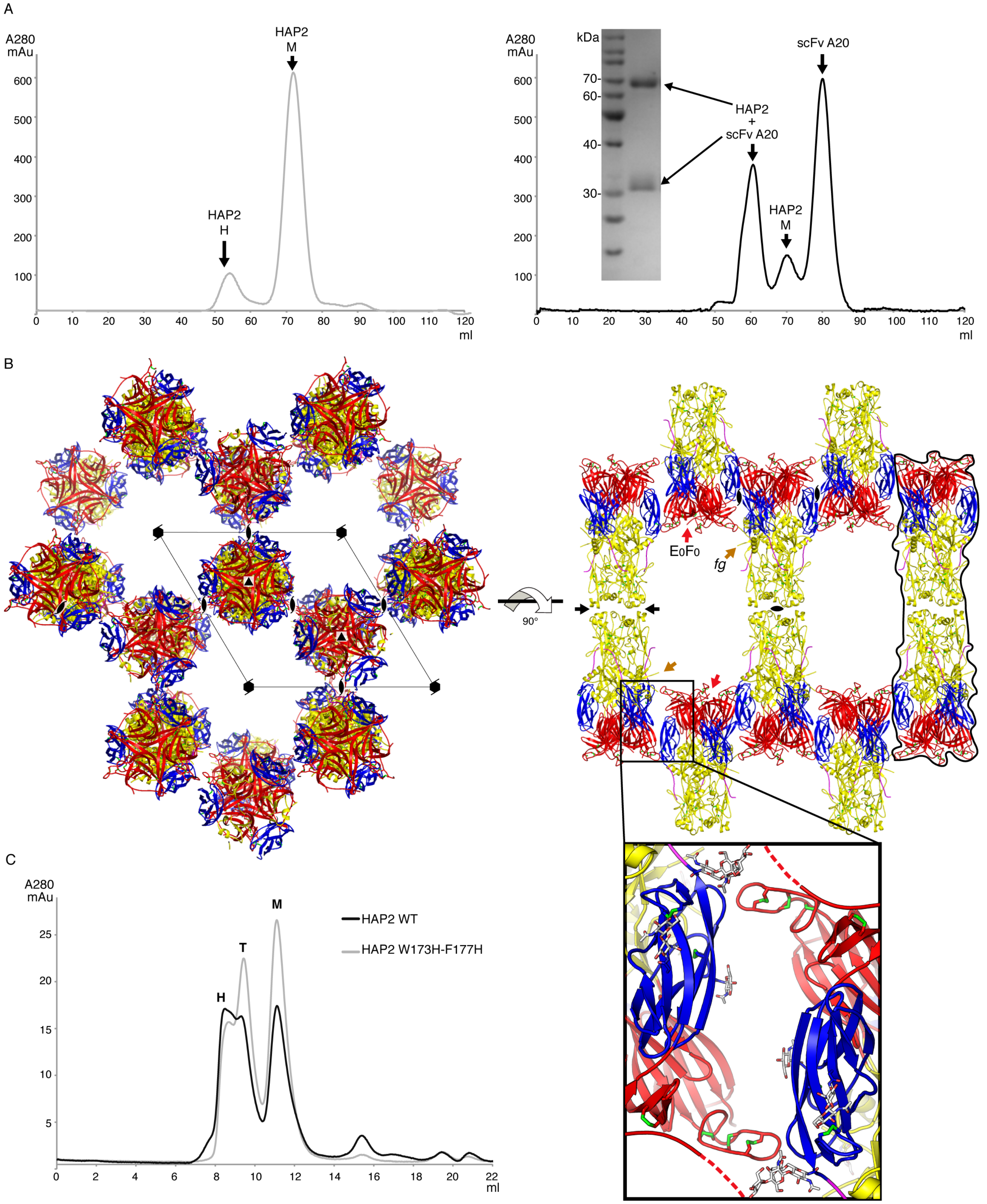

Supplemental Figure S2

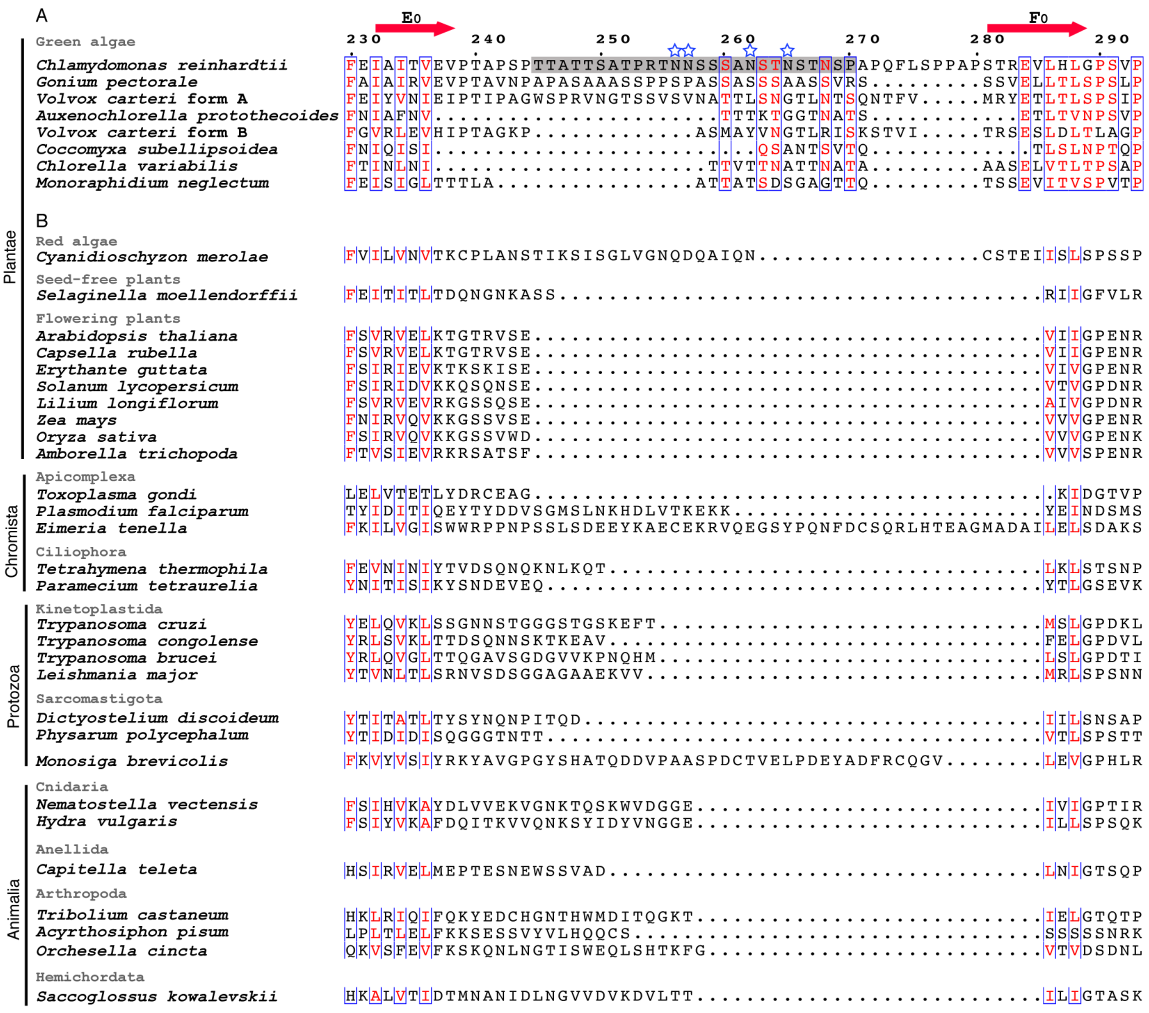

Supplemental Figure S3

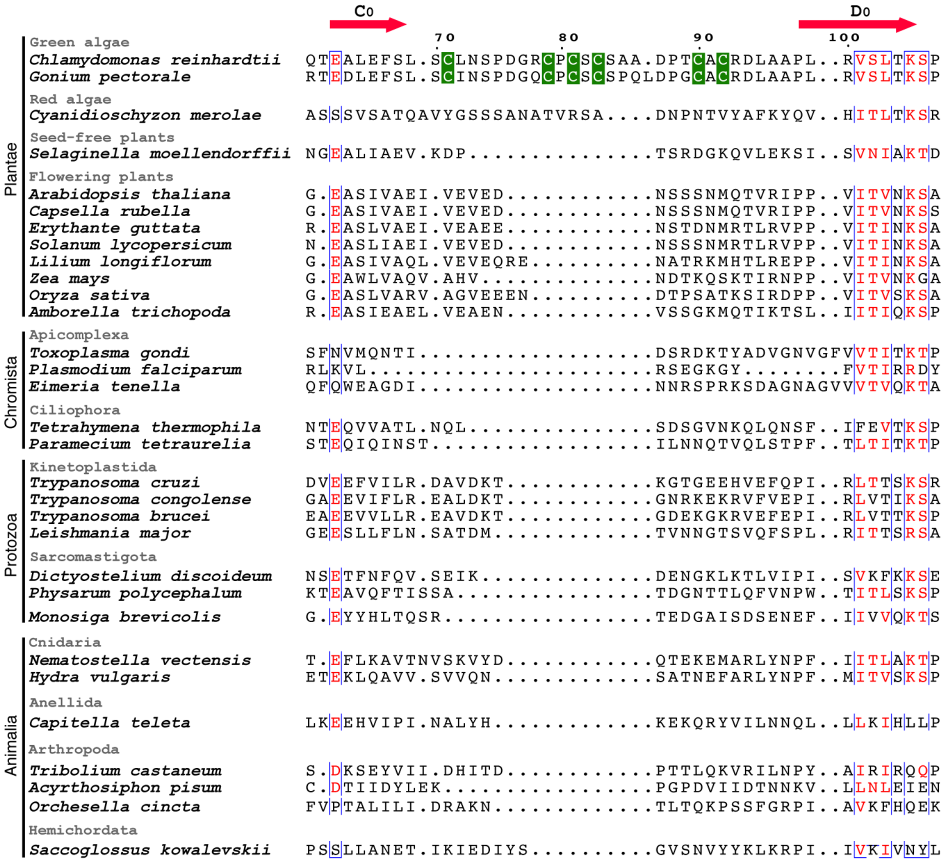

Supplemental Figure S4

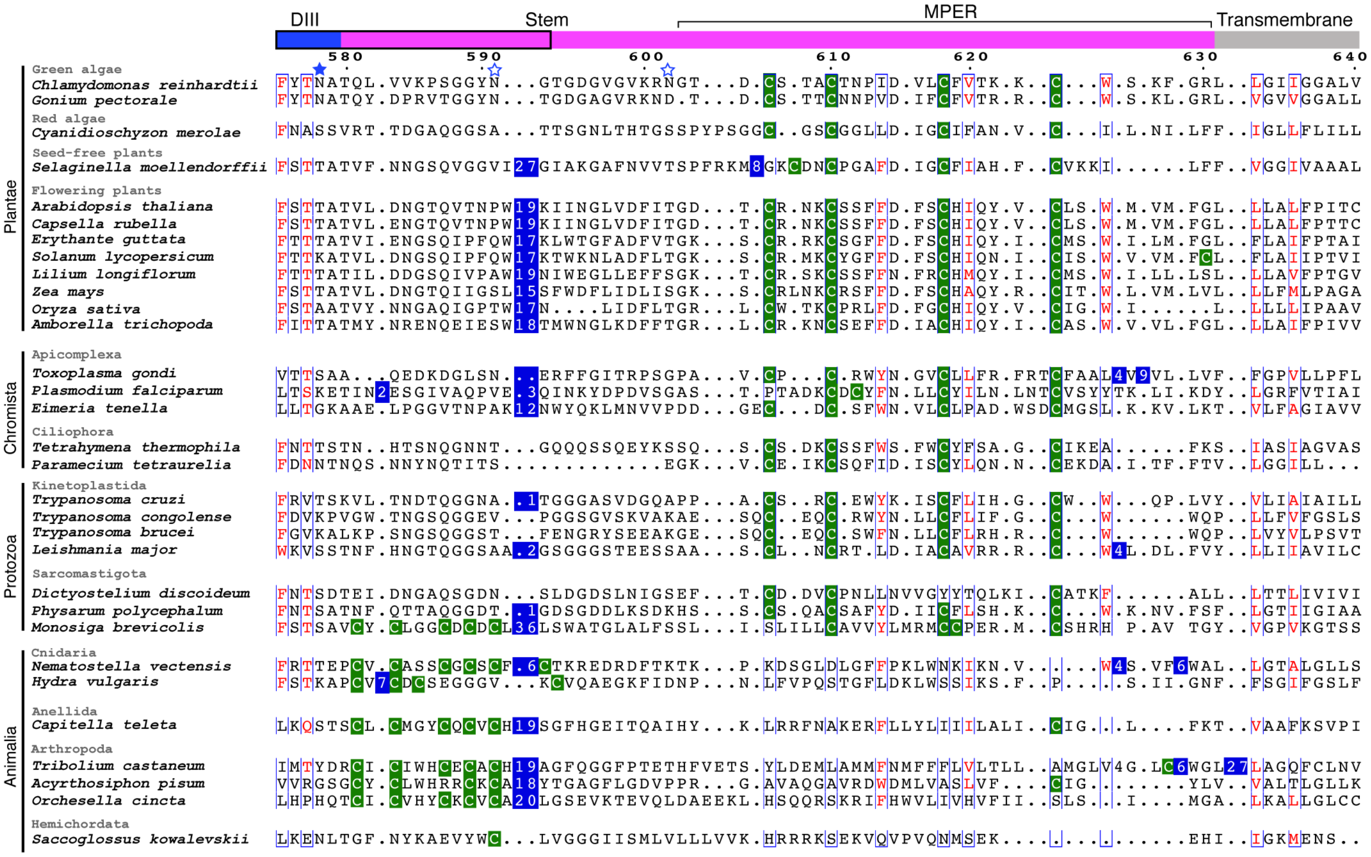

Supplemental Figure S5

A

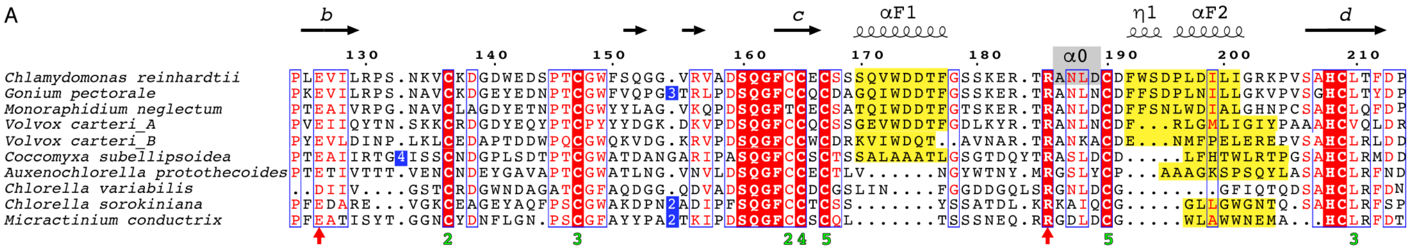

B

Helix αF1

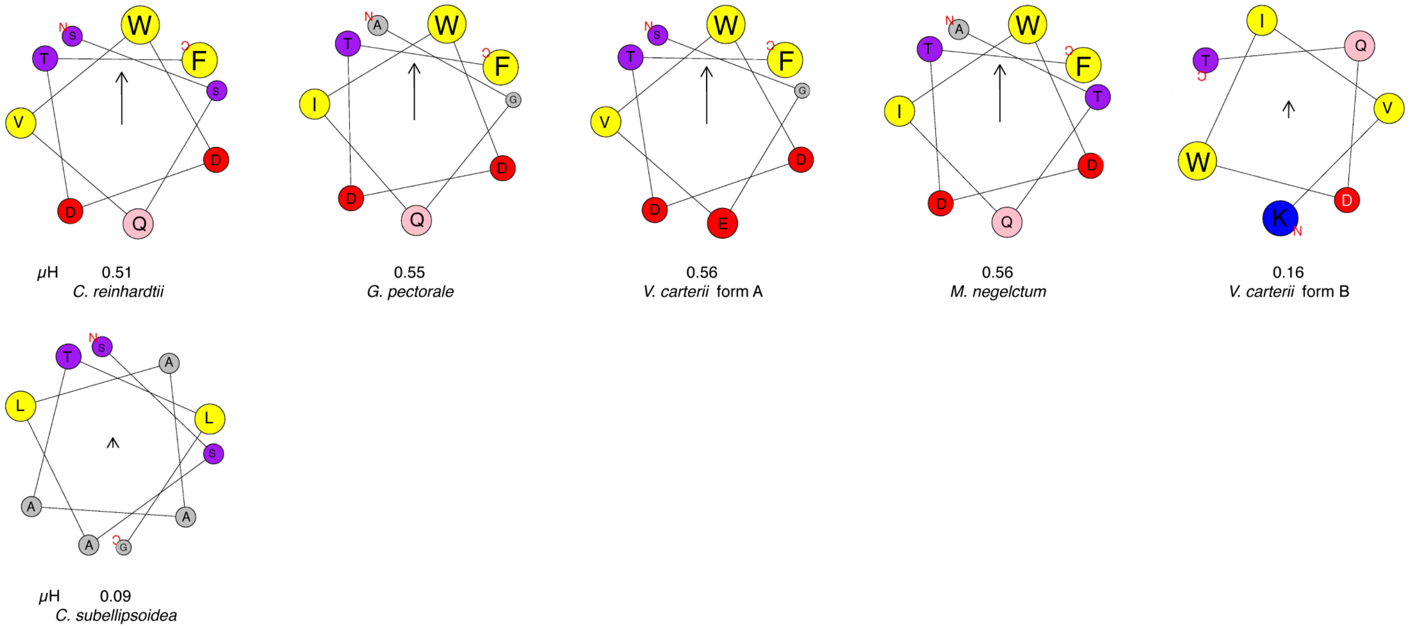

Helices ηF and αF2

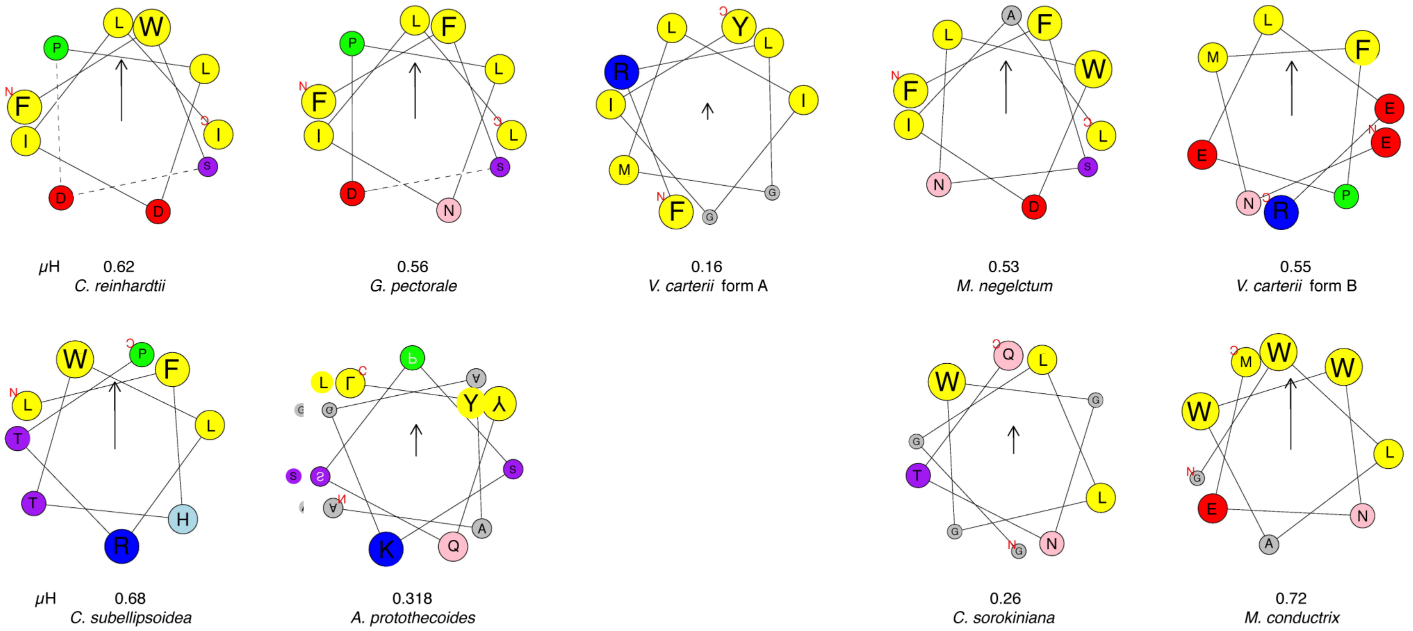

Supplement: Document S1. Figures S1–S5 [file mmc1.pdf]
